# The CORE-KDT study: a mixed methods protocol to establish core outcomes for refractory childhood epilepsy treated with ketogenic diet therapy

**DOI:** 10.1186/s13063-022-06629-7

**Published:** 2022-08-17

**Authors:** Jennifer H. Carroll, J. Helen Cross, Mary Hickson, Emma Williams, Valerie Aldridge, Avril Collinson

**Affiliations:** 1 Faculty of Health, University of Plymouth, Plymouth, Devon UK; 2 UCL Developmental Neurosciences, UCL - NIHR BRC Great Ormond Street Institute of Child Health, London, UK; 3 Matthew’s Friends, Lingfield, Surrey, UK

**Keywords:** Core outcome set, Delphi survey, Epilepsy, Ketogenic diet, Outcomes, Paediatric, Systematic scoping review, Semi-structured interview, Consensus method

## Abstract

**Background:**

A core outcome set defines the minimum outcomes that should be included in clinical trials, audit or practice. The aim being to increase the quality and relevance of research by ensuring consistency in the measurement and reporting of outcomes. Core outcome sets have been developed for a variety of disease states and treatments. However, there is no established set of core outcomes for refractory childhood epilepsy treated with ketogenic diet therapy. This should be developed using a patient-centred approach to ensure the outcomes measured are relevant to patients and clinical practice.

**Methods:**

This is a mixed methods study of four phases to develop a core outcome set for refractory childhood epilepsy treated with ketogenic diet therapy. In phase 1, a systematic scoping review of the literature will establish which outcomes are measured in trials of refractory epilepsy treated with ketogenic diet therapy. In phase 2, qualitative interviews with parents and carers will aim to identify the outcomes of importance to these stakeholders. Phase 3 will see a comprehensive list of outcomes collated from the first two phases, grouped into domains according to an outcome taxonomy. Phase 4 will invite parents, health care professionals and researchers to participate in a two-round Delphi study to rate the importance of the presented outcomes. Following which, the core outcome set will be ratified at a face to face consensus meeting.

**Discussion:**

This study will guide outcome measurement in future studies of childhood epilepsy treated with ketogenic diet therapy and clinical practice through audit and service evaluation.

## Background

Epilepsy is a common neurological disorder where up to one third of children become drug resistant or refractory [1], experiencing regular debilitating seizures, despite treatment with multiple antiepileptic medications. When medication fails to control seizure activity, non-pharmacological treatments such as ketogenic diet (KD) therapy are considered.

The KD is a very low carbohydrate and high fat regimen, used to treat refractory epilepsy since the 1920s [2]. It mimics a starvation state whereby the bodies main energy source switches from that of glucose to ketones produced through lipolysis of high levels of dietary fat. KD therapy is a well-established treatment for refractory epilepsy with a growing number of randomised controlled trials demonstrating efficacy [3–10]. Yet the exact anticonvulsant mechanism is not clear [11]. When treating epilepsy, National Institute for Health and Care Excellence (NICE) guidance suggests seizure freedom as a primary outcome and secondary outcomes should include seizure reduction, quality of life and cognitive function [12]. Yet in published clinical effectiveness trials, seizure reduction and/or freedom are typically the primary outcomes with side effects of treatment often assessed as secondary outcomes [13]. Less frequently considered are health-related quality of life outcomes such as reduced hospitalisation [14], medication load and cost [15], improved behaviour and cognition [16, 17].

KD therapy is a resource-intensive treatment requiring regular input and monitoring from a team of specialists, including a ketogenic dietitian and paediatric neurologist. For the family, it is often a labour-intensive regimen that requires significant dietary adjustment for the child. Whilst more recently developed KDs offer improved palatability and reduced potential for adverse side effects, adherence to the dietary regimen may not always be easy. When successful it can have a significant impact on functioning and quality of life [16] for the child and wider family, yet such outcomes are inconsistently measured and reported between trials. The development of a core outcome set is one method proposed to address these problems.

A core outcome set defines the minimum outcomes that should be consistently measured and reported in future clinical trials in a specific area of healthcare [18]. A core outcome set would reduce outcome reporting bias, drive up quality and relevance of research, improve reporting consistency and support meta-analysis leading to better informed healthcare decision making [19]. It would also serve to guide outcome assessment in clinical practice through audit and service evaluation. Successful examples of core outcome sets include Outcome Measures in Rheumatology (OMERACT) [20]; the Initiative on Methods, Measurement and Pain Assessment in Clinical Trials (IMMPACT) [21]; and Harmonising Outcome Measures in Eczema (HOME) [22].

The most recent Cochrane review [23] concluded that a core outcome set would help to improve consistency in outcomes for drug resistant epilepsy treated with ketogenic diet. Core outcome sets are developed using consensus methods in partnership with major stakeholders, including experts in the clinical area, patients and parents where appropriate [18]. This patient-centred approach will ensure outcomes are clinically relevant and reflect the views of parents and carers. Previous studies have examined parental expectations [24, 25] and attitudes [26] towards KD therapy via questionnaires, but no attempts have been made to establish parental opinion on outcomes of importance.

## Aims and objectives

### Aim

The overall aim of this project is to develop a core outcome set for refractory childhood epilepsy treated with KD therapy. The study will identify the outcomes to be measured in clinical effectiveness trials but will also guide audit or service evaluation in clinical practice. Parents^1^, health care professionals, researchers, relevant charities and industry will be consulted to ensure the final core outcome set reflects the interests of all and facilitates future decision making.

### Objectives

The key objectives are as follows: (1) to identify a list of outcomes from published studies using KD therapy to treat childhood epilepsy, (2) to identify the tools or methods used to measure the reported outcomes, (3) to determine a list of potentially important outcomes to parents of a child with epilepsy treated with KD therapy and (4) to collate the outcomes identified in (1) and (3) and reach consensus on a core outcome set from the perspective of parents and healthcare professionals.

## Methods

The study is registered with The Core Outcome Measures in Effectiveness Trials (COMET) Initiative (#1116) [27] and will follow its procedures and guidance [18]. Ethical approval was granted by the National Health Service (NHS) Health Research Authority (London-Surrey Research Ethics Committee, reference 19/LO/1680). Written informed consent will be gathered from participants. The study will be divided into four distinct phases. Phase 1 will identify a list of all possible relevant outcomes and the tools used to measure these, via a systematic scoping review of studies involving children with epilepsy treated with KD therapy. Phase 2 will undertake semi-structured interviews with up to 20 parents who have a child with epilepsy treated with KD therapy, in order to identify potential additional outcomes important to them. Phase 3 will define outcome domains into which outcomes, identified in the scoping review and qualitative interviews, will be grouped according to the COMET taxonomy [28]. Phase 4 will prioritise the most important outcomes from two stakeholder groups via a two-arm anonymous remote Delphi survey. Stakeholder group 1 will include health professionals and researchers and group 2 will include parents. Few children would have the understanding or capacity to participate in this study so the researchers have elected to interview parents only. The findings of this work will be integrated into a core outcome set at a consensus group meeting with representation from both stakeholder groups.

### Public involvement

The importance of involving families in research is well documented [29, 30]. From the outset, we have recognised the value and importance of parents and carers as stakeholders and worked closely with our lay research partners (EW and VA) at Matthew’s Friends, a charity supporting families with KD therapies, to guide the design and delivery of the CORE-KDT study. A patient and public involvement consultation was undertaken with recruitment supported by Young Epilepsy, a charity for children and young people with epilepsy, and Matthew’s Friends. Two parents with children with epilepsy on KD therapy were interviewed. They felt this study of outcomes was worthwhile research and welcomed the inclusion of parents as participants in each phase. The findings informed the design of the semi-structured interview schedule for use in phase 2 and highlighted that the main considerations when undertaking interviews with parents are likely to be time and competing demands. It was felt parents are more likely to choose a telephone or video call for ease and convenience instead of a face-to-face meeting.

A study advisory group will be convened involving both health professionals and parents of children with epilepsy. Representatives from relevant UK charities will be consulted (Young Epilepsy and Matthew’s Friends), the latter playing a particular role in supporting families to undertake KD therapy. This group will provide oversight for the study and review key documentation such as, but not limited to, participant information and the semi-structured interview script. In addition, they will participate in the phase 3 consultation process to ratify the list of outcomes arising from phases 1 and 2 and associated lay descriptors in preparation for the 2-round Delphi study.

### Phase 1: Systematic scoping review of outcomes measured and reported for childhood epilepsy treated with ketogenic diet therapy

Research question: what outcomes are measured and reported in studies of childhood epilepsy treated with ketogenic diet therapy?

#### Search strategy

The proposed scoping review is registered on the Joanna Briggs Institute Systematic Review Register [31] and the detailed protocol agreed a priori and published [32]. In summary, the proposed review will be conducted in accordance with the Preferred Reporting Items for Systematic Reviews and Meta-Analyses extension for Scoping Reviews (PRISMA-ScR) [33]. An initial limited search of CINAHL and PubMed will be undertaken to identify key search terms and inform the development of a tailored search strategy for each information source. The extensive search strategy will aim to identify both published and unpublished studies. Reference lists of systematic reviews will be reviewed to ensure all primary studies have been identified. Reference lists of included full-text articles will be hand searched for additional studies. CINAHL, MEDLINE, Cochrane Database of Systematic Reviews, Cochrane CENTRAL, Embase, AMED, Scopus and Joanna Briggs Institute Evidence Synthesis will be searched. Trial registers including ClinicalTrials.gov and International Standard Randomised Controlled Trials Number (ISRCTN) Registry will be checked. Unpublished grey literature will sought via OpenGrey (System for Information on Grey Literature in Europe SIGLE) OAIster and British Library e-theses (EThOS). Search results will be catalogued in Endnote V8 (Clarivate Analytics, PA, USA) reference manager.

#### Types of studies

There are a limited number of randomised controlled trials examining KD therapy so clinical trials and observational studies published in English will be included. Searches will be undertaken over a 10 year period, as the wide scoping nature of this review is likely to identify a large number of studies for inclusion within which repetition of measured and reported outcomes is expected. The potential for omission of outcomes of importance will be ameliorated by offering participants the opportunity to identify other outcomes of importance in the semi-structured qualitative interviews (parents) and Delphi study (parents, health professionals and researchers).

#### Type of intervention

A single intervention: KD therapy is under investigation. Ketogenic diets are high fat, very low carbohydrate and adequate protein diets. KD therapy encompasses all types of ketogenic diet used in clinical trials and practice namely, classical KD, medium chain triglyceride (MCT) KD, the Modified Atkins diet, modified ketogenic diet therapy and low glycaemic index treatment. Participants may be treated with other medical therapies or surgery in conjunction with KD therapy.

#### Types of participants

Studies of male or female children under the age of 18 years old with refractory epilepsy treated with KD therapy for at least 1 month.

#### Exclusion criteria

Studies of children treated with KD therapy for a diagnosis other than epilepsy (for example metabolic disease and neuro-oncology) and studies of adult participants.

#### Eligibility of studies

Two reviewers (JC and a researcher with significant experience in systematic review methods) will independently assess the title and abstracts returned from searches to assess whether the papers meet the inclusion criteria. Where it is unclear from the abstract then the full text will be retrieved and assessed. Authors will be contacted to request full-text access where necessary. Full-text studies that do not meet the inclusion criteria will be excluded and reasons for exclusion stated. If agreement regarding eligibility cannot be reached, a third reviewer within the research team will be consulted. Study protocols will be requested from authors of included studies to compare reporting of outcomes in study protocol with those reported in the final publication.

#### Data extraction

Data will be extracted by one reviewer from the full text of original studies using a pre-defined and piloted spreadsheet. A second reviewer will independently extract data from 10% of included studies, chosen at random, to check for consistency. As a minimum, the following data will be extracted; journal of publication and year, study type, author details, participant characteristics, intervention (variant of KD), outcomes reported, definition of outcome, the tool or indicators used to measure the outcome, the validity of assessment tools used and the frequency of outcome measurement.

#### Data analysis and presentation

The scoping review protocol and subsequent report will follow the PRISMA-ScR process [33]. A PRISMA [34] flowchart will outline the stages of the systematic search. The extracted data will be presented using tables and figures to best meet the objectives of the scoping review. A narrative summary will follow with discussion of the key findings. The final list of identified outcomes will be used in phase 3 of this study.

### Phase 2: Semi-structured interviews with parents of a child with epilepsy treated with ketogenic diet therapy

Research question: What outcomes do parents regard as potentially important when undertaking ketogenic diet therapy for the treatment of refractory childhood epilepsy?

### Overview and method

The objective of this qualitative description study is to establish which outcomes are valued by parents and carers. It is recommended that patients and the public be consulted when developing a core outcome set, preserving the perspective of these stakeholders and improving the accessibility of the later consensus process for participants [18, 35]. Parent proxy reporting is an accepted approach when the child is unable to respond independently, for example, due to age, cognitive impairment or illness [36]. Few children would have the understanding or capacity to participate in this study so the researchers have elected to interview parents only. Data generated through qualitative research is accepted to be contextually rich and meaningful, enabling an in-depth exploration of issues that cannot be achieved through quantitative methods alone [37]. Interestingly, core outcome set studies which sought patient or public opinion highlighted further outcomes of importance that were not previously identified through systematic review of published studies [38–40]. Stratified purposeful sampling will be used to assess a range of perspectives on the topic under investigation. A sampling frame will be used to monitor the clinical and socio-demographic characteristics of participants to ensure diversity in terms of the following characteristics: age of child, diagnosis, type of KD, duration of treatment with KD therapy and response to treatment. Parental experiences of a recently diagnosed infant who has just commenced KD therapy will likely differ from those whose adolescent child is diagnosed many years and stable on KD therapy. It is plausible that these differing experiences may influence the identification and perceived importance of outcomes. Therefore, a range of ages will be included from infant (0–2 years), young child (2–6 years), child (6–12 years) to adolescent (12–18 years) [41], within which there is expected to be variety in the duration of treatment and response to KD therapy. This will be broadly defined as recently commenced KD therapy (≤ 3 months of treatment with KD therapy), established on KD therapy (4 months or longer) or weaned from KD therapy (in the previous 12 months). The sampling frame will be assessed iteratively as recruitment proceeds and advertising materials refocussed to seek under-represented groups if necessary. We aim to recruit 20 parents, although this may change depending on early analyses. Other notable studies [42, 43] reached saturation at between 15 and 16 participants where no new outcomes were being identified and further interviews would provide no new additional insights [37]. A semi-structured interview script will be prepared and piloted with lay patient research partners. All interviews will be audio recorded and conducted by the primary researcher (JC).

#### Participants

Parent participants will be invited through clinical partners at NHS Trusts, relevant UK charities (Matthew’s Friends, Young Epilepsy and Epilepsy Action), and ‘Epilepsy – The Ketogenic Way’ Facebook group. International participants will be reached via the aforementioned charities and Facebook group. Participants can register their interest on our study webpage [44] and access participant information regarding the details of the study. Informed written consent will be sought prior to the interview.

#### Inclusion criteria

Parents of a child aged 0–18 years with refractory epilepsy who is currently being treated with KD therapy or has weaned from KD in the past year and is able to participate in an interview in the English language.

#### Exclusion criteria

Parents of a child being treated with KD therapy for a condition other than epilepsy (for example metabolic disease and neuro-oncology). Parents of a child previously treated with KD therapy but who weaned from the diet over 1 year ago. Inability to understand the English language.

#### Interview format and data collection

Interviews will be undertaken by JC, a registered dietitian and researcher with approximately 12 years’ experience with KD therapy. A semi-structured interview format will be used. A conversational style of interviewing using open questions will encourage a naturalistic account of parent’s experiences and perspectives on topics such as epilepsy diagnosis, treatment with KD and the effect of these on their child and family (semi-structured interview schedule available in the Table 2 in Appendix). Outcomes will be identified by asking participants to identify in their opinion, the important outcomes for children with epilepsy treated with KD therapy. Participants who list multiple outcomes will be asked to prioritise, to help us to understand the outcomes they value most. Alone, this approach might result in a narrow view on outcomes, identifying only those outcomes that parents understand to be results or outcomes. To mitigate this, outcomes will also be identified indirectly via a content analysis of the full interview transcripts. Together, this will enable all possible outcomes to be identified.

Interviews with UK participants will be undertaken in a convenient location such as the family home, video or audio call. Interviews with international participants will be undertaken via video or audio call. There is a possibility that this method may reduce rapport and recognition of non-verbal cues [45] but others [46] argue it is comparable to in person face to face interviews. Despite these potential challenges, video conferencing technology enables the inclusion of otherwise inaccessible international participants to this study. Written consent will be taken prior to the interviews and participants were reminded that they can stop the interview or withdraw from the study at any point.

#### Analysis of semi-structured interviews

A reflective research diary will be used to document reflections and findings post interview to support later analysis. Audio recordings of the interviews will be fully transcribed, stored and analysed using NVivo software (QSR International, Burlington, MA, USA). A content analysis will be undertaken to identify new outcomes, not previously identified in the systematic scoping review of literature. A further thematic analysis will explore parent’s experiences of epilepsy and KD therapy [47, 48]. The aim being to identify the outcomes in the narrative materials and to identify common threads that extend across the set of interviews. The analytical process will begin during data collection with the first two interviews being transcribed and analysed to enable iterative changes to the interview schedule and ongoing data collection. Codes will be generated from the data and modified to accommodate new data and insights. The study team can then refine questions, develop hypotheses and pursue emerging avenues of inquiry further in subsequent interviews. Coding and identification of themes will be conducted by the lead researcher JC in collaboration with a senior researcher experienced in qualitative research methods, who will independently review 10% of the coded transcripts. The final themes and newly identified outcomes will be agreed by all authors through discussion. Newly identified outcomes will be added to the database derived from the scoping review.

### Phase 3: Consultation process

Research question: What outcomes should be entered into a Delphi process for further study?

#### Overview and method

The combined list of potential outcomes derived from the systematic review in phase 1 and semi-structured interviews with parents in phase 2 will be grouped into outcome domains according to an outcome taxonomy (Table 1) [28]. This is an updated version of Williamson & Clarks original taxonomy [18] which was developed following review of two cohorts of Cochrane systematic reviews [49, 50] and the outcomes recommended in 198 core outcome sets [51]. The findings will be presented to the research team and advisory panel for review. Any disagreement will be discussed and resolved. The purpose being to ratify the list of outcomes, ensuring consistent, accessible language and definitions, whilst avoiding duplication.

**Table 1:** Outcome Taxonomy adapted from Dodd et al. [28]

**Outcome taxonomy**	
1. Mortality	
2. 2–24: **Physiological/clinical**	
2: Blood and lymphatic system outcomes	
3: Cardiac outcomes	
4: Congenital, familial and genetic outcomes	
5: Endocrine outcomes	
6: Ear and labyrinth outcomes	
7: Eye outcomes	
8: Gastrointestinal outcomes	
9: General outcomes	
10: Hepatobiliary outcomes	
11: Immune system outcomes	
12: Infection and infestation outcomes	
13: Injury and poisoning outcomes	
14: Metabolism and nutrition outcomes	
15: Musculoskeletal and connective tissue outcomes	
16: Outcomes relating to neoplasms: benign, malignant and unspecified	
17: Nervous system outcomes	
18: Pregnancy, puerperium and perinatal outcomes 19: Renal and urinary outcomes	
20: Reproductive system and breast outcomes	
21: Psychiatric outcomes	
22: Respiratory, thoracic and mediastinal outcomes	
23: Skin and subcutaneous tissue outcomes	
24: Vascular outcomes	
**Functioning**	
25: Physical functioning	
26: Social functioning	
27: Role functioning	
28: Emotional functioning/well-being	
29: Cognitive functioning	
31: Perceived health status	
32: Delivery of care, including;	
- Satisfaction/patient preference	
- Acceptability and availability	
- Adherence/compliance	
- Withdrawal from treatment	
- Appropriateness of treatment	
- Process, implementation, and service outcomes	
33: Personal circumstances	
**Resource use**	
34: Economic	
35: Hospital	
36: Need for further intervention	
37: Societal/carer burden	
38: Adverse events/effects	

### Phase 4: Prioritisation of outcomes according to stakeholder group and integration of outcomes into a core outcome set

Research question: What are the most important outcomes to include in a core outcome set for refractory childhood epilepsy treated with ketogenic diet therapy?

### Overview and method

A survey of key stakeholders will be undertaken using Delphi survey methodology following recommended practices in the development of core outcome sets [18]. An online questionnaire will rate the importance of the outcomes identified in phase three. This questionnaire will be developed and administered using DelphiManager software. Representatives from two stakeholder groups will be asked to pilot the survey prior to dissemination to all participants (group one, health professionals and researchers; group two, parents). Participants will be invited to rate each outcome in two Delphi rounds, with high scores indicating the importance of inclusion in the final core outcome set.

#### Stakeholders

Parent participants will be invited through clinical partners at NHS Trusts, relevant UK charities (Matthew’s Friends, Young Epilepsy and Epilepsy Action), and ‘Epilepsy – The Ketogenic Way’ Facebook group. International participants will be reached via the aforementioned charities and Facebook group. Health and neurology professionals (e.g. paediatric neurologists, paediatricians, ketogenic dietitians, epilepsy specialist nurses, clinical and educational psychologists) will be invited to participate through specialist interest groups and professional societies (e.g. British Paediatric Neurology Association, Ketogenic Professional Advisory Group, Ketogenic Dietitians Research Network, Epilepsy Nurses Association, Neurological and Neuropsychology special interest groups of the British Psychology Society, NHS regional Paediatric Epilepsy Network and Cochrane Epilepsy). Industry representatives with relevant experience with ketogenic diet therapy will also be invited. International colleagues will be invited through professional networks. The study will be presented at relevant conferences and meetings to raise awareness and aid recruitment. Participants can register their interest by contacting the research team or visiting the study website [44] to access the appropriate participant information for each stakeholder group.

#### Survey administration

There are no recommendations for appropriate sample sizes for Delphi surveys. We will therefore be guided by other relevant Delphi surveys [43, 52, 53] and aim to recruit between 20–50 participants in each stakeholder group within the available timeframe. Potential participants will be asked to register through an online platform or by contacting the research team. Whilst the use of KD therapy has grown exponentially over the past decade, there are estimated to be only 750 patients in the UK on KD therapy with 250 waiting to commence therapy [54]. We will aim for representation across a range of age groups, epilepsy diagnosis, duration of treatment with KD therapy and type of KD therapy. Informed consent will be assumed if participants register online for the Delphi survey and submit their answers. The age of the child undertaking KD therapy will be recorded, time on KD, diagnosis, ethnicity and the country of residence.

There are approximately 100 paediatric neurologists nationally in the UK [52] not all of whom will have experience with KD therapy and approximately 90 ketogenic dietitians. The small size of the UK health professional group means that international recruitment is essential. The inclusion of international health professionals and researchers will also ensure that the outcome core set is acceptable worldwide. We will aim for optimal diversity through representation of as many of the aforementioned health professionals in the professional stakeholder group. Profession, experience with KD therapy and country in which they practice will be recorded. Informed consent will be assumed if participants register online for the Delphi survey and submit their answers. Each participant will be assigned a unique identifier to ensure anonymity, yet enable the research team to monitor their participation and send invitation and reminder emails. The COMET initiative DelphiManager software will be used to administer the survey. Two Delphi rounds will be undertaken in line with other core outcome set studies [55–57] as three rounds may be overly burdensome on participants. Equally, two rounds are expected to be sufficient given the focussed nature of the single intervention (KD therapy) under investigation.

#### Delphi survey round one

Participants will be asked to identify which stakeholder group they belong to using a dropdown menu and to complete additional demographic questions. Health professionals and researchers will identify their profession, country of work and experience with KD therapy. Parents will identify their child’s diagnosis, age, duration of treatment with KD therapy and type of KD. All participants will be asked to complete the round one survey within 3 weeks. They will be prompted at the end of week two with a reminder email if the survey has not yet been completed. The survey will be identical for both stakeholder groups*.* Prior to commencing the Delphi survey and rating each presented outcome individually, participants will be asked to blindly list five outcomes that are most important to them. They will then proceed to the Delphi survey and rate the importance of each outcome identified in phase three*.* A 9-point Likert scoring system will be used in line with other core outcome set studies [43, 53, 57] where 1–3 signifies an outcome is of limited importance, 4–6 important but not critical and 7–9 is of critical importance. An ‘unable to score’ option will be included for stakeholders who may not have the expertise to score all outcomes. Partial responses will be included. A final free text section will encourage participants to list any other outcomes they feel are not represented in the survey but are of importance. These will be considered for inclusion in round 2.

#### Delphi survey round one analysis

Descriptive statistics will summarise the aggregate results of round one for each stakeholder group. Differences between health professional responses (e.g. ketogenic dietitians compared to paediatric neurologists) will be assessed. The feasibility of which depends on the number of respondents from each health profession represented.

#### Delphi survey round two

Respondents to the round one survey will be invited to participate in round two. All outcomes will be carried forward from round one and any new outcomes potentially identified through the free text question in round one. Participants will be reminded of their own individual score for each outcome and see the aggregate scores of both stakeholder groups. Participants will be asked to reflect on their answer and re-score again the importance of each outcome. They will be encouraged to explain their rationale for any changes via a free text box. Presenting the aggregate scores for each stakeholder group has been shown to improve consensus between groups in what is important to retain in the final core outcome set [58]. A final question will ask the respondent if they would like to attend the face to face consensus meeting.

#### Delphi survey round 2 analysis and defining consensus

Descriptive statistics will summarise the aggregate results of round 2 for each stakeholder group. To define consensus, the survey responses will be analysed separately for each stakeholder group. A 70/15% consensus definition is proposed [18, 59, 60] whereby an outcome is included in the core outcome set if >70% of each stakeholder group rated it 7–9 and <15% considered it of little importance by scoring it 1–3. Finally, there may be outcomes where there is only partial or no agreement between stakeholder groups that warrant further discussion at the final consensus group meeting.

#### Consensus group meeting

A face to face meeting will be convened at a relevant conference to improve accessibility and attendance. An equal number of each stakeholder group will be randomly chosen from those who identified a willingness to attend the meeting. Participants will be supported to attend. Results for all outcomes will be presented along with the draft core outcome set. Stakeholders will take part in facilitated small group discussion to consider outcomes that did not reach consensus in the Delphi survey. Anonymous remote voting will be utilised. Outcomes will be included in the final set if 70% of voters score the outcome between 7 and 9.

#### Dissemination

Ultimately our goal is to develop a core set of outcomes that will aid consistency in outcome measurement and reporting in future trials and clinical practice. However, its use will likely be limited if too many outcomes are included. A working group including members of the research team and expert stakeholders will be formed to explore ways to measure the agreed outcomes and support dissemination. If the resultant core outcome set is too large, the working group will aim to refine it further, ensuring it is practical for use, whilst still preserving the views and insights of the wider stakeholders identified during the interviews, Delphi study and consensus meeting. The final core outcome set will be reported following the Core Outcome Set - Standards for Reporting (COS-STAR) statement and checklist [61]. Dissemination will occur via engagement with trialists, Cochrane, COMET, and publication in relevant journals. Study participants who opted to receive study updates will be sent a newsletter and links to relevant publications.

## Discussion

Summarised here is the protocol of a mixed methods study to develop a core outcome set. This will guide outcome measurement and reporting in future trials of refractory childhood epilepsy treated with KD therapy. Professional networks regularly highlight the lack of consensus in outcome collection as an area for development. The findings will therefore inform and support clinicians undertaking audit and service evaluation. It might be argued that KD therapy as a treatment for refractory epilepsy is a niche area affecting a relatively small group of patients and the need for a core outcome set questioned. However, a core outcome set is indicated when considering the complexity of refractory epilepsy, the difficulties in achieving seizure control, the unique and intensive nature of KD therapy and the challenges families face when caring for a child with significant health needs. A core outcome set for self limited epilepsy with centro temporal spikes, an epilepsy limited to childhood,  was recently published [62] and whilst there are likely to be some shared outcomes when both are compared, it is expected that our proposed set may capture different or additional outcomes relevant to the complexity of refractory epilepsy and severity of associated co-morbidities. These might include epilepsy-related hospital admissions, antiepileptic drug reduction, financial burden and adverse effects of KD therapy. The collaborative and patient-centred approach, with parent involvement throughout will ensure the agreed core outcomes reflect the views of all major stakeholders. Two key challenges for core outcome set developers include achieving global consensus and implementation of the finalised core outcome set in future clinical trials [59]. To address these, the researchers will engage with international partners early in the study to foster participation and engagement. Expert panels at key conferences and engagement in professional networks will support this. Finally, the researchers will actively engage with trialists, regulators and funding bodies to ensure the finalised core outcome set is recognised and used.

### Trial status

Version 1.4 protocol November 2020. This study is not a trial. Participant recruitment for the qualitative interviews and Delphi study will begin in January 2020.

## Data Availability

The dataset generated during this study will be available from the University of Plymouth on reasonable request.

## Appendix

Table 2

**Table 2:** Semi-structured interview schedule

1.	Please start by telling me the story of your child’s epilepsy
2.	Could you tell me how your child’s epilepsy has affected you and your family?
3.	Thinking back to before your child started ketogenic diet, can you tell me what your expectations or hopes of the diet were?
4.	Were those expectations delivered? (what has changed with ketogenic diet?)
5.	Can I ask, how did that make you feel?
6.	Has that changed - do you still feel that way now?
7.	As you are aware we are interested in the results or outcomes that parents believe are important to assess in clinics and research, what results do you think are important when using the KD?
8.	If you were asked to prioritise, what would be the most important result or outcome?
9.	Can you tell me about the day-to-day management of the KD?
10.	What might help to make KD easier for families?
11.	Do you think a buddy or mentoring programme would be helpful where parents support each other with KD?

## Abbreviations

COMET: Core Outcome Measures in Effectiveness Trials
COS-STAR: Core Outcome Set - Standards for Reporting
HOME: Harmonising Outcome Measures in Eczema
IMMPACT: The Initiative on Methods, Measurement and Pain Assessment in Clinical Trials
ISRCTN: International Standard Randomised Controlled Trials Number Registry
JBI: Joanna Briggs Institute
KD: Ketogenic diet
MCT: Medium chain triglyceride
NHS: National Health Service
NICE: National Institute for Health and Care Excellence
OMERACT: Outcome Measures in Rheumatology
PRISMA: Preferred Reporting Items for Systematic Reviews and Meta-analyses
RCT: Randomised controlled trail
SIGLE: System for Information on Grey Literature in Europe

## Footnotes

1 The term ‘parent’ will be used throughout and includes carers and legal guardians.

## References

[1] Kwan P, Schachter SC, Brodie MJ (2011). Drug-resistant epilepsy. N Engl J Med.

[2] Wilder RM (1921). The effects of ketonemia on the course of epilepsy. Mayo Clin Proc.

[3] Neal EG, Chaffe H, Schwartz RH, Lawson MS, Edwards N, Fitzsimmons G (2008). The ketogenic diet for the treatment of childhood epilepsy: a randomised controlled trial. Lancet Neurol.

[4] Sharma S, Sankhyan N, Gulati S, Agarwala A (2013). Use of the modified Atkins diet for treatment of refractory childhood epilepsy: a randomized controlled trial. Epilepsia..

[5] Lambrechts DA, de Kinderen RJ, Vles JS, de Louw AJ, Aldenkamp AP, Majoie HJ (2017). A randomized controlled trial of the ketogenic diet in refractory childhood epilepsy. Acta Neurol Scand.

[6] Raju KN, Gulati S, Kabra M, Agarwala A, Sharma S, Pandey RM (2011). Efficacy of 4:1 (classic) versus 2.5:1 ketogenic ratio diets in refractory epilepsy in young children: a randomized open labeled study. Epilepsy Res.

[7] Kim JA, Yoon JR, Lee EJ, Lee JS, Kim JT, Kim HD (2016). Efficacy of the classic ketogenic and the modified Atkins diets in refractory childhood epilepsy. Epilepsia..

[8] Sharma S, Goel S, Jain P, Agarwala A, Aneja S (2016). Evaluation of a simplified modified Atkins diet for use by parents with low levels of literacy in children with refractory epilepsy: a randomized controlled trial. Epilepsy Res.

[9] El-Rashidy OF, Nassar MF, Abdel-Hamid IA, Shatla RH, Abdel-Hamid MH, Gabr SS (2013). Modified Atkins diet vs classic ketogenic formula in intractable epilepsy. Acta Neurol Scand.

[10] Seo JH, Lee YM, Lee JS, Kang HC, Kim HD (2007). Efficacy and tolerability of the ketogenic diet according to lipid:nonlipid ratios-comparison of 3:1 with 4:1 diet. Epilepsia..

[11] Freeman J, Veggiotti P, Lanzi G, Tagliabue A, Perucca E (2006). The ketogenic diet: from molecular mechanisms to clinical effects. Epilepsy Res.

[12] National Institute for Health and Care Excellence (NICE) (2016). Epilepsies: diagnosis and management. Clinical Guideline [CG137].

[13] Martin-Mcgill KJ, Jackson CF, Breshnahan R, Levy RG, Cooper PN (2018). Ketogenic diet for drug-resistant epilepsy. Cochrane Database Syst Rev.

[14] Joshi M, Kearns J, Wilford E, Hussain N, Khan A (2016). Effectiveness of ketogenic diet to reduce seizure related acute paediatric admission in children with Epilepsy. Dev Med Child Neurol.

[15] Gilbert DL, Pyzik PL, Vining EPG, Freeman JM (1999). Medication cost reduction in children on the ketogenic diet: data from a prospective study. J Child Neurol.

[16] IJff DM, Postulart D, Lambrechts DAJE, Majoie MHJM, de Kinderen RJA, Hendriksen JGM (2016). Cognitive and behavioral impact of the ketogenic diet in children and adolescents with refractory epilepsy: a randomized controlled trial. Epilepsy Behav.

[17] Hallböök T, Ji S, Maudsley S, Martin B (2012). The effects of the ketogenic diet on behavior and cognition. Epilepsy Res.

[18] Williamson PR, Altman DG, Bagley H, Barnes KL, Blazeby JM, Brookes ST (2017). The COMET Handbook: version 1.0. Trials..

[19] Williamson P, Altman D, Blazeby J, Clarke M, Gargon E (2012). Driving up the quality and relevance of research through the use of agreed core outcomes. J Health Serv Res Policy.

[20] Boers M, Kirwan JR, Wells G, Beaton D, Gossec L, d'Agostino MA (2014). Developing core outcome measurement sets for clinical trials: OMERACT Filter 2.0. J Clin Epidemiol.

[21] Turk DC, Dworkin RH, Allen RR, Bellamy N, Brandenburg N, Carr DB (2003). Core outcome domains for chronic pain clinical trials: IMMPACT recommendations. Pain..

[22] Schmitt J, Apfelbacher C, Spuls PI, Thomas KS, Simpson EL, Furue M (2015). The Harmonizing Outcome Measures for Eczema (HOME) roadmap: a methodological framework to develop core sets of outcome measurements in dermatology. J Invest Dermatol.

[23] Martin-McGill KJ, Bresnahan R, Levy RG, Cooper PN (2020). Ketogenic diets for drug-resistant epilepsy (Review). Cochrane Database Syst Rev.

[24] Farasat S, Kossoff EH, Pillas DJ, Rubenstein JE, Vining EP, Freeman JM (2006). The importance of parental expectations of cognitive improvement for their children with epilepsy prior to starting the ketogenic diet. Epilepsy Behav.

[25] Bruce S, Devlin A, Air L, Cook L (2017). Changes in quality of life as a result of ketogenic diet therapy: a new approach to assessment with the potential for positive therapeutic effects. Epilepsy Behav.

[26] Schoeler NE, MacDonald L, Champion H, Cross JH, Sander JW, Sisodiya SM (2014). Assessing parents’ attitudes towards ketogenic dietary therapies. Epilepsy Behav.

[27] COMET. 2019 [Available from: http://www.comet-initiative.org/].

[28] Dodd S, Clarke M, Becker L, Mavergames C, Fish R, Williamson PR (2018). A taxonomy has been developed for outcomes in medical research to help improve knowledge discovery. J Clin Epidemiol.

[29] INVOLVE. Briefing notes for researchers: involving the public in NHS, public health and social care research. Eastleigh INVOLVE; 2012.

[30] Rosenbaum P (2011). Family-centered research: what does it mean and how do we do it?. Dev Med Child Neurol.

[31] Joanna Briggs Institute. Systematic Review Register 2020. Available from: https://joannabriggs.org/systematic-review-register. (Accessed on 23 June 2020)

[32] Carroll J, Martin-McGill K, Cross H, Hickson M, Collinson A (2019). Outcome measurement and reporting in childhood epilepsy treated with ketogenic diet therapy: a scoping review protocol. JBI Database System Rev Implement Rep.

[33] Tricco AC, Lillie E, Zarin W, O'Brien KK, Colquhoun H, Levac D (2018). PRISMA Extension for Scoping Reviews (PRISMA-ScR): checklist and explanation. Ann Intern Med.

[34] Moher D, Liberati A, Tetzlaff J, Altman DG (2010). Preferred reporting items for systematic reviews and meta-analyses: the PRISMA statement. Int J Surg.

[35] Young B, Bagley H (2016). Including patients in core outcome set development: issues to consider based on three workshops with around 100 international delegates. Res Involv Engagem.

[36] Ronen GM, Streiner DL, Rosenbaum P (2003). Health-related quality of life in children with epilepsy: development and validation of self-report and parent proxy measures. Epilepsia..

[37] Mack N, Woodsong C, MacQueen K, Guest G, Namey E (2005). Qualitative research methods: a data collectors field guide.

[38] Rosenbaum SE, Glenton C, Nylund HK, Oxman AD (2010). User testing and stakeholder feedback contributed to the development of understandable and useful Summary of Findings tables for Cochrane reviews. J Clin Epidemiol.

[39] Arnold LM, Crofford LJ, Mease PJ, Burgess SM, Palmer SC, Abetz L (2008). Patient perspectives on the impact of fibromyalgia. Patient Educ Couns.

[40] Kirwan J, Heiberg T, Hewlett S, Hughes R, Kvien T, Ahlmen M (2003). Outcomes from the patient perspective workshop at OMERACT 6. J Rheumatol.

[41] World Health Organisation (2007). Paediatric age categories to be used in differentiating between listing on a model essential medicines list for children. Position Paper. USA.

[42] MacLennan S, Williamson PR, Bekema H, Campbell M, Ramsay C, N'Dow J (2017). A core outcome set for localised prostate cancer effectiveness trials. BJU Int.

[43] Murugupillai R, Wanigasinghe J, Muniyandi R, Arambepola C (2016). Parental concerns towards children and adolescents with epilepsy in Sri Lanka-Qualitative study. Seizure..

[44] Carroll J (2019). Core outcomes for refractory childhood epilepsy treated with ketogenic diet therapy. The Core-KDT study.

[45] Iacono VL, Symonds P, Brown DHK (2016). Skype as a tool for qualitative research interviews.

[46] Deakin H, Wakefield K (2013). Skype interviewing: reflections of two PhD researchers.

[47] Braun V, Clarke V (2006). Using thematic analysis in psychology. Qual Res Psychol.

[48] Vaismoradi M, Turunen H, Bondas T (2013). Content analysis and thematic analysis: Implications for conducting a qualitative descriptive study. Nurs Health Sci.

[49] Smith V, Clarke M, Williamson P, Gargon E (2015). Survey of new 2007 and 2011 Cochrane reviews found 37% of prespecified outcomes not reported. J Clin Epidemiol.

[50] Davey J, Turner RM, Clarke MJ, Higgins JP (2011). Characteristics of meta-analyses and their component studies in the Cochrane Database of Systematic Reviews: a cross-sectional, descriptive analysis. BMC Med Res Methodol.

[51] Gargon E, Gurung B, Medley N, Altman D, Blazeby J, Clarke M (2014). Choosing important health outcomes for comparative effectiveness research: a systematic review. PLoS One.

[52] Morris C, Dunkley C, Gibbon FM, Currier J, Roberts D, Rogers M (2017). Core Health Outcomes In Childhood Epilepsy (CHOICE): protocol for the selection of a core outcome set. Trials..

[53] Murugupillai R, Ranganathan SS, Wanigasinghe J, Muniyandi R, Arambepola C (2018). Development of outcome criteria to measure effectiveness of antiepileptic therapy in children. Epilepsy Behav.

[54] Whitely VJ, Martin-McGill KJ, Carroll JH, Taylor H, Schoeler N (2020). on behalf of the Ketogenic Dietitians Research Network (KDRN). Nice to know: impact of NICE guidelines on ketogenic diet services nationwide. J Hum Nutr Diet.

[55] Sinha IP, Gallagher R, Williamson PR, Smyth RL (2012). Development of a core outcome set for clinical trials in childhood asthma: a survey of clinicians, parents, and young people. Trials..

[56] Blazeby JM, Macefield R, Blencowe NS, Jacobs M, McNair AGK, Sprangers M (2015). Core information set for oesophageal cancer surgery. Br J Surg.

[57] Harman NL, Bruce IA, Kirkham JJ, Tierney S, Callery P, O'Brien K (2015). The importance of integration of stakeholder views in core outcome set development: otitis media with effusion in children with cleft palate. PLoS One.

[58] Brookes ST, Macefield RC, Williamson PR, McNair AG, Potter S, Blencowe NS (2016). Three nested randomized controlled trials of peer-only or multiple stakeholder group feedback within Delphi surveys during core outcome and information set development. Trials..

[59] Williamson PR, Altman DG, Blazeby JM, Clarke M, Devane D. Developing core outcome sets for clinical trials: issues to consider. Trials. 2012;13(132):919–26. 10.1186/1745-6215-13-132 PMC3472231 22867278

[60] Wylde V, MacKichan F, Bruce J, Gooberman-Hill R (2015). Assessment of chronic post-surgical pain after knee replacement: development of a core outcome set. Eur J Pain.

[61] Kirkham JJ, Gorst S, Altman DG, Blazeby JM, Clarke M, Devane D (2016). Core Outcome Set-STAndards for Reporting: the COS-STAR statement. PLoS Med.

[62] Crudgington H, Rogers M, Bray L, Carter B, Currier J, Dunkley C (2019). Core Health Outcomes in Childhood Epilepsy (CHOICE): Development of a core outcome set using systematic review methods and a Delphi survey consensus. Epilepsia..

